# Elevation of Mac‐2 binding protein glycosylation isomer after hepatectomy is associated with post‐hepatectomy liver failure, total Pringle time, and renal dysfunction

**DOI:** 10.1002/ags3.12271

**Published:** 2019-07-08

**Authors:** Daisuke Imai, Takashi Maeda, Huanlin Wang, Kensaku Sanefuji, Hiroto Kayashima, Shohei Yoshiya, Kazuki Takeishi, Shinji Itoh, Noboru Harada, Toru Ikegami, Tomoharu Yoshizumi, Masaki Mori

**Affiliations:** ^1^ Department of Surgery Hiroshima Red Cross Hospital & Atomic Bomb Survivors Hospital Hiroshima Japan; ^2^ Department of Surgery and Science Graduate School of Medical Sciences Kyushu University Fukuoka Japan

**Keywords:** liver resection, Mac‐2 binding protein glycosylation isomer, post‐hepatectomy liver failure, Pringle maneuver, renal dysfunction

## Abstract

**Background:**

Mac‐2 binding protein glycosylation isomer (M2BPGi) is a novel serum glycomarker used to assess liver fibrosis. However, it has been reported that M2BPGi is likely to reflect other factors not limited to liver fibrosis.

**Methods:**

We retrospectively analyzed 79 patients with liver tumors who underwent liver resection. M2BPGi was measured within 1 week before operation and almost 1 month after operation. We introduced a value termed the “ΔM2BPGi ratio” (=M2BPGi_after operation_/M2BPGi_before operation_), and analyzed factors that influenced the ΔM2BPGi ratio.

**Results:**

The median value of the ΔM2BPGi ratio was 1.28 (range, 0.36‐5.68). In 64 patients (81.0%), the cutoff index values of M2BPGi were elevated approximately 1 month after operation, especially in patients who experienced post‐hepatectomy liver failure (PHLF). Multiple linear regression showed total Pringle time, PHLF grade ≥B, and preoperative value of creatinine were significant predictors of the ΔM2BPGi ratio. The mean values of the ΔM2BPGi ratio were 1.37 ± 0.07, 1.52 ± 0.22, and 2.94 ± 0.30 for PHLF grade 0, grade A, and grade B, respectively, resulting in statistically significant differences by the Kruskal‐Wallis test (*P *=* *0.022).

**Conclusions:**

Total Pringle time, PHLF grade ≥B, and preoperative creatinine significantly influenced the elevation of M2BPGi almost 1 month after liver resection. This study strongly affirms the previous suggestion that M2BPGi is likely to reflect other factors not limited to liver fibrosis.

## 1 INTRODUCTION

Mac‐2 binding protein glycosylation isomer (M2BPGi) is a novel serum glycomarker used to assess liver fibrosis in patients infected with chronic hepatitis C^1^, ^2^, ^3^ or B virus^4^, ^5^ as well as in patients with primary biliary cirrhosis,^6^, ^7^ biliary atresia,^8^ autoimmune hepatitis,^9^ and non‐alcoholic fatty liver disease.^10^, ^11^, ^12^

Although M2BPGi level correlates with liver fibrosis, M2BPGi levels immediately decrease following the eradication of hepatitis C virus (HCV) by direct‐acting antiviral therapy, whereas the rapid improvement of liver fibrosis beyond 24 weeks post‐treatment is unlikely.^13^ Furthermore, M2BPGi was increased in patients with acute liver injury and decreased after their recovery.^14^ These observations suggest that M2BPGi reflects liver fibrosis and other factors such as liver inflammation, liver damage, and hepatocyte regeneration.

In this study, we measured serum M2BPGi levels in patients who underwent liver resection for liver tumors before and approximately 1 month after operation. Our aims were to analyze which factors influence perioperative changes in the M2BPGi value, and to evaluate the biological features of M2BPGi.

## 2 MATERIALS AND METHODS

### 2.1 Patients

Between December 2016 and December 2018, 120 consecutive patients underwent liver resection for liver tumor at Hiroshima Red Cross Hospital & Atomic Bomb Survivors Hospital. Of these, patients who underwent open microwave ablation without liver resection (four) and patients with a lack of pre‐ or postoperative M2BPGi test results (33) were excluded from this study. Four patients who underwent postoperative M2BPGi testing over 1 month after operation were also excluded for data accuracy. Consequently, 79 patients were eligible for this study.

A preoperative serum sample was collected at the time of admission for liver resection within 1 week before surgery, and a postoperative sample was collected at the outpatient clinic approximately 1 month after liver resection. M2BPGi values were measured at SRL (Tokyo, Japan), and reported as the cutoff index (COI). We introduced a value termed the “ΔM2BPGi ratio” (=M2BPGi_after operation_/M2BPGi_before operation_) for perioperative changes in the M2BPGi value. We also analyzed predictors of the ΔM2BPGi ratio. Other serologic fibrosis markers, 7S domain of type IV collagen (4COL7S) and hyaluronic acid were also measured at SRL.

Post‐hepatectomy liver failure (PHLF) was diagnosed on the basis of the International Study Group of Liver Surgery definition.^15^ Briefly, elevated prothrombin time – international normalization ratio and concomitant hyperbilirubinemia on or after postoperative day 5 was defined as PHLF. The severity of PHLF was graded as follows: Grade A, PHLF that required no change in a patient's clinical management; Grade B, PHLF that required a deviation from the regular course but did not require invasive therapy; and Grade C, PHLF that required invasive treatment including intensive care.

The study protocol was approved by our institutional ethics committee.

### 2.2 Statistical analysis

All statistical analyses were performed using SAS software (JMP 13.0.1; SAS Institute Inc., Cary, NC, USA). Continuous variables were expressed as the means ± standard deviations or medians with ranges, and compared using Student's *t*‐test. Categorical variables were compared using the Chi‐square test or Fisher exact test as required. The difference among PHLF grades and between paired stages was compared using the Kruskal‐Wallis test and the Wilcoxon rank‐sum test, respectively. In multiple linear regression, predictors were selected through a stepwise procedure using the minimum Bayesian information criterion (BIC) method among the factors selected in single linear regression.

## 3 RESULTS

### 3.1 Patient characteristics

Patient characteristics, tumor status, and summary of the postoperative course are summarized in Table 1. The study population consisted of 55 men and 24 women with a median age of 72 years (range, 36‐91). Among these patients, 30 (38.0%) were positive for HCV antibody, 22 (27.8%) were positive for HBV surface antigen, and 27 (34.2%) were negative for both (nonBnonC). The Child‐Pugh score was 5 in 59 (74.7%) patients, 6 in 16 (20.3%) patients, and 7 in four (5.1%) patients.

**Table 1:** Patient demographic and clinical characteristics

Factors	Total cases (n = 79)
Sex, male (%)	55 (69.6)
Age, years	72 (36‐91)
Etiology (%)	
HCV	30 (38.0)
HBV	22 (27.8)
nonBnonC	27 (34.2)
Child‐Pugh score (%)	
5	59 (74.7)
6	16 (20.3)
7	4 (5.1)
Operative factors	
Operative time (min)	224 (111‐488)
Total Pringle time (min)	49 (0‐150)
Blood loss (mL)	234 (0‐2227)
Blood transfusion (%)	4 (5.0)
Liver resection (%)	
Partial hepatectomy	34 (43.0)
Subsegmentectomy	34 (43.0)
Sectionectomy	8 (10.1)
Lobectomy	3 (3.8)
Pathological findings (%)	
HCC	59 (74.7)
Combined HCC‐CCA	6 (7.6)
ICC	6 (7.6)
Metastatic tumor	4 (5.0)
Benign tumor	4 (5.0)
Tumor size (cm)	2.2 (0.7‐13)
Single tumor (%)	58 (75.3)
*F* factor (%)	
0	6 (7.8)
1	4 (6.5)
2	13 (16.9)
3	17 (20.8)
4	37 (48.1)
Postoperative course	
Postoperative hospital stay, days	12 (7‐30)
Clavien‐Dindo classification (%)	
0	49 (63.6)
I	15 (19.5)
II	12 (15.6)
III	0
IV	1 (1.3)
V	0
Post‐hepatectomy liver failure (%)	
0	68 (86.1)
A	7 (8.9)
B	4 (5.0)
C	0

Abbreviations: CCA, cholangiocarcinoma; HBV, hepatitis B virus; HCC, hepatocellular carcinoma; HCV, hepatitis C virus; ICC, intrahepatic cholangiocarcinoma.

The median operative time was 224 minutes (range, 111‐488), with a median total Pringle time of 49 minutes (range, 0‐150) and median blood loss of 234 g (range 0‐2247). Blood transfusion was performed in four (5.0%) patients. The type of hepatectomy included nonanatomical partial resection and subsegmentectomy in 34 (43.0%) patients each, sectionectomy in eight (10.1%), and lobectomy in three (3.8%).

The pathological diagnosis of the liver tumor was hepatocellular carcinoma (HCC) in 59 patients (74.7%), combined hepatocellular cholangiocellular carcinoma and intrahepatic cholangiocarcinoma in six each, and metastatic tumor and benign tumor in four each. The median diameter of the largest tumor was 2.2 cm (range, 0.7‐13), and the median tumor number was one (range, 1‐3). Pathological F4 was observed in 37 patients (48.1%).

The median length of postoperative hospital stay was 12 days (range, 7‐30). Twenty‐eight (46.4%) patients experienced postoperative complications: Clavien‐Dindo Class I in 15 (19.5%) patients, Class II in 12 (15.6%), and Class IV in one (1.3%). PHLF occurred in 11 patients (13.9%): Grade A in seven (8.9%) and Grade B in four (3.0%).

### 3.2 Performance of M2BPGi as a marker for liver fibrosis

**Figure 1:**
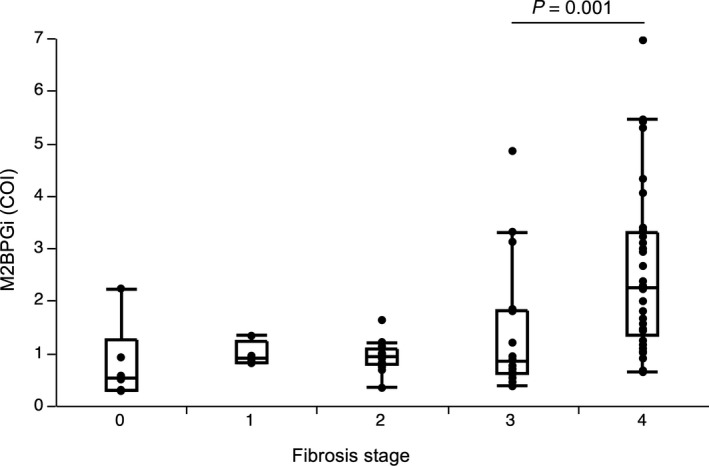
Mac‐2 binding protein glycosylation isomer (M2BPGi) values for each liver fibrosis stage

The mean COI value of preoperative M2BPGi was 1.78 ± 1.41. The M2BPGi level was positively correlated with pathological fibrosis stage (Figure 1). The mean COI values were 0.80 ± 0.51, 0.98 ± 0.62, 0.94 ± 0.35, 1.39 ± 0.30, and 2.55 ± 0.20 in stages F0, F1, F2, F3, and F4 patients, respectively, resulting in a statistically significant difference by Kruskal‐Wallis test (*P *<* *0.001). Differences between the abutting stages were significant between F3 and F4 (*P *=* *0.001, Wilcoxon rank‐sum test).

### 3.3 The relationship between pre‐ and postoperative M2BPGi

**Figure 2:**
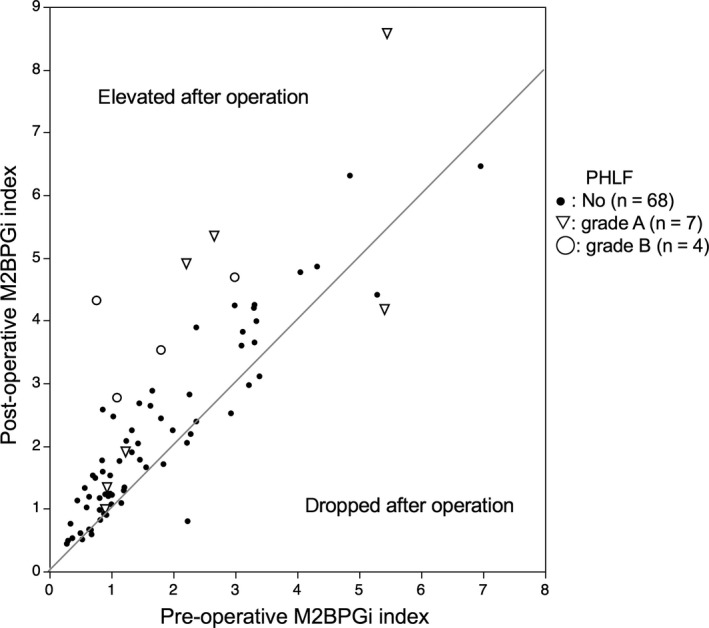
Correlation between pre‐ and postoperative Mac‐2 binding protein glycosylation isomer (M2BPGi)

The mean postoperative M2BPGi value was 2.32 ± 1.63. The median number of days between operation and serum sampling was 22 days (range, 18‐29). The relationship between pre‐ and postoperative M2BPGi is shown in Figure 1. The postoperative M2BPGi values were strongly correlated to preoperative values (*R* ^2^ = 0.77, *P *<* *0.001). The median value of the ΔM2BPGi ratio was 1.28 (range 0.36‐5.68). In 64 patients (81.0%), the M2BPGi values were elevated after operation, especially in patients who experienced PHLF (Figure 2).

In addition, it should be noted that we had one patient whose M2BPGi value markedly dropped after liver resection (Figure 2). Her preoperative COI value of M2BPGi was 2.23 and dropped to 0.8 after operation (ΔM2BPGi ratio = 0.36). The patient was 72 years old and underwent S8 partial hepatectomy for colorectal liver metastases. Her preoperative liver function was normal with Child‐Pugh score of 5; indocyanine green retention rate at 15 minutes, 21.2; platelet count, 11.2 × 10^4^/μL. Her renal function was also normal with 0.6 mg/dL of serum creatinine. She was negative for HCV‐Ab or HBsAg. The operative time was 192 minutes and total blood loss was 486 g. Pringle maneuver could not be performed due to her history of multiple hepatectomies. Maximum tumor size was 2.5 cm and the number of tumors was three. Pathological analysis revealed F0 fibrosis and A1 inflammation of background liver. Her postoperative course was good without any complications.

### 3.4 Factors that influence the ΔM2BPGi ratio

Univariate and multivariate analyses of perioperative parameters to predict the value of the ΔM2BPGi ratio were performed. A simple linear regression was calculated to predict the ΔM2BPGi ratio based on each factor as shown in Table 2. A significant regression equation was found for females (*F* = 5.31, *P *=* *0.024, *R* ^2^ = 0.05), creatinine (*F* = 5.16, *P *=* *0.026, *R* ^2 ^= 0.07), blood loss (*F* = 4.77, *P *=* *0.033, *R* ^2 ^= 0.05), operative time (*F* = 19.3, *P *<* *0.001, *R* ^2 ^= 0.19), total Pringle time (*F* = 21.0, *P *<* *0.001, *R* ^2 ^= 0.21), PHLF grade ≥B (*F* = 26.3, *P *<* *0.001, *R* ^2 ^= 0.24), complication of Clavien‐Dindo Class ≥II (*F* = 6.91, *P *=* *0.010, *R* ^2 ^= 0.07), and anatomical liver resection (*F* = 5.73, *P *=* *0.019, *R* ^2 ^= 0.07). These eight factors were included in a stepwise procedure (minimum BIC method) to select variables for multiple linear regression analysis. Creatinine, total Pringle time and PHLF grade ≥B were selected, and a multiple linear regression was calculated to predict the ΔM2BPGi ratio based on these three factors. A significant regression equation was found (*F* = 17.1, *P *<* *0.0001) where *R* ^2^ *= *0.40. The patient predicted ΔM2BPGi ratio = 1.536 + 0.0054 × total Pringle time (minutes) + 0.2760 × creatinine (mg/dL) + 0.656 × PHLF grade≥ B, where PHLF grade ≥B was coded as 1 = Yes, −1 = No. Creatinine (*P *=* *0.013), total Pringle time (*P *=* *0.002) and PHLF grade ≥B (*P *<* *0.001) were all significant predictors of the ΔM2BPGi ratio.

**Table 2:** Univariate and multivariate analyses: risk factors associated with increased post/preoperative M2BPGi ratio (n = 79)

Factors	Univariate analysis			Multivariate analysis		
	B	SE B	*β*	B	SE B	*β*
Sex, female	−0.19	0.08	−0.25*	NA		
Age	<−0.001	0.006	−0.01			
HCV	0.08	0.08	0.11			
nonBnonC	−0.05	0.08	−0.07			
Creatinine (mg/dL)	0.30	0.13	0.26*	0.28	0.11	0.24*
Child‐Pugh score	−0.04	0.22	−0.02			
FIB‐4 index	−0.01	0.03	−0.04			
Tumor size (cm)	−0.02	0.04	−0.02			
Single tumor	0.14	0.09	0.18			
Liver cirrhosis (F4)	−0.12	0.08	−0.18			
Blood loss (mL)	<0.001	<0.001	0.24*	NA		
Operative time (min)	0.003	<0.001	0.45***	NA		
Blood transfusion	−0.25	0.17	−0.16			
Total Pringle time (min)	0.007	0.001	0.47***	0.005	0.001	0.31**
PHLF grade ≥B	−0.77	0.15	−0.40***	−0.66	0.15	−0.43***
Clavien‐Dindo class ≥II	−0.26	0.10	−0.29*	NA		
Anatomical resection	−0.18	0.08	−0.26*			
The number of days Operation—Check of postoperative M2BPGi	−0.03	0.03	−0.11			

Abbreviations: HCV, hepatitis C virus; NA, not available; PHLF, post‐hepatectomy liver failure; SE, standard error.

*P <* *0.05.

**P <* *0.01.

***P <* *0.001.

**Figure 3:**
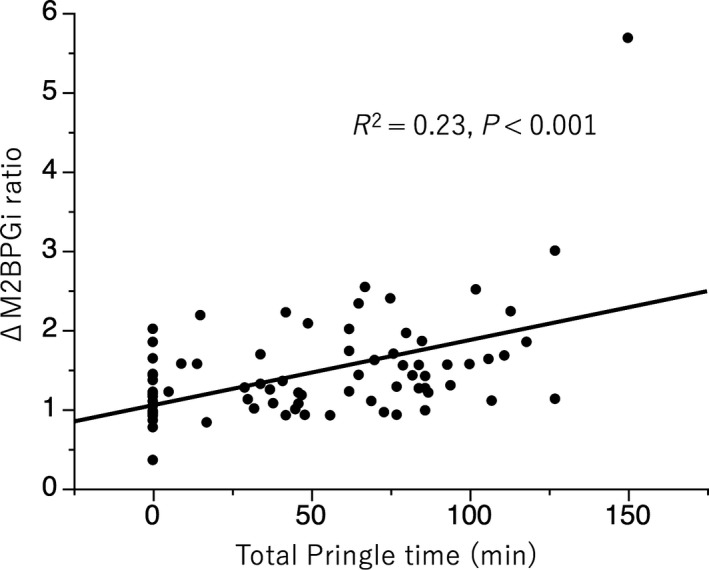
Relationship between ΔM2BPGi ratio and total Pringle time

We evaluated the association between ΔM2BPGi ratio and total Pringle time in more detail (Figure 3). The total Pringle time was weakly correlated to ΔM2BPGi ratio (*R* ^2 ^= 0.23, *P *<* *0.001).

### 3.5 Relationship between PHLF and serologic fibrosis markers

**Figure 4:**
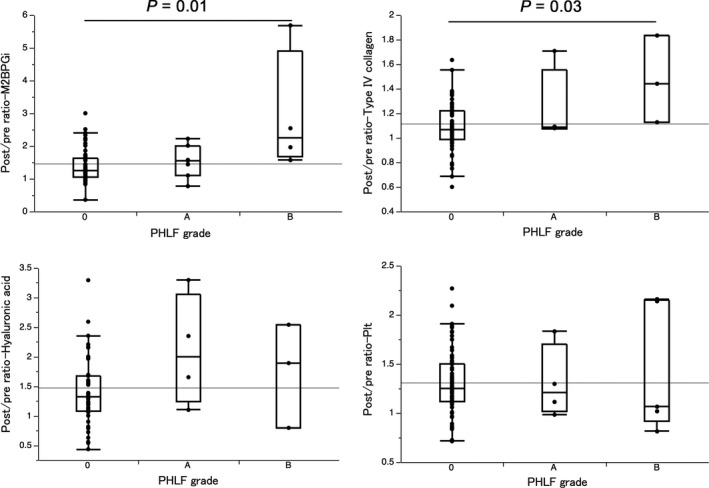
Relationship between post‐hepatectomy liver failure and serologic fibrosis markers

The mean values of the ΔM2BPGi ratio were 1.37 ± 0.07, 1.52 ± 0.22, and 2.94 ± 0.30 for PHLF grade 0, grade A, and grade B, respectively, resulting in statistically significant differences by the Kruskal‐Wallis test (*P *=* *0.022, Figure 4). Differences between the paired stages were significant between grades 0 and B (*P *=* *0.008; Wilcoxon rank‐sum test). We examined relationships between PHLF and the other fibrosis markers. We also introduced the ΔHyaluronic acid ratio (=Hyaluronic acid_after operation_/Hyaluronic acid_before operation_), Δ4COL7S ratio (=4COL7S_after operation_/4COL7S_before operation_), and ΔPlt ratio (=Plt_after operation_/Plt_before operation_) to determine changes in the value around the time of operation. The mean values were as follows. ΔHyaluronic acid ratio: 1.41 ± 0.08, 1.88 ± 0.27, 1.74 ± 0.35; Δ4COL7S ratio: 1.09 ± 0.03, 1.16 ± 0.09, 1.47 ± 0.12; and ΔPlt ratio: 1.30 ± 0.04, 1.16 ± 0.13, 1.53 ± 0.18, for PHLF grade 0, grade A, and grade B, respectively. None of these demonstrated a statistically significant difference by Kruskal‐Wallis test. However, differences in the Δ4COL7S ratio between the paired PHLF stages were significant between grades 0 and B (*P *=* *0.033; Wilcoxon rank‐sum test).

## 4 DISCUSSION

The present study is the first to investigate predictive factors for the elevation of M2BPGi after liver resection. Contrary to our expectations, the M2BPGi values were elevated in 80% of all cases even approximately 1 month after liver resection. Furthermore, the factors that influenced the elevation of M2BPGi after liver resection were preoperative creatinine, PHLF grade ≥B and total Pringle time. These observations confirm the previous suggestion that M2BPGi is likely to reflect other factors not limited to liver fibrosis.^13^

**Figure 5:**
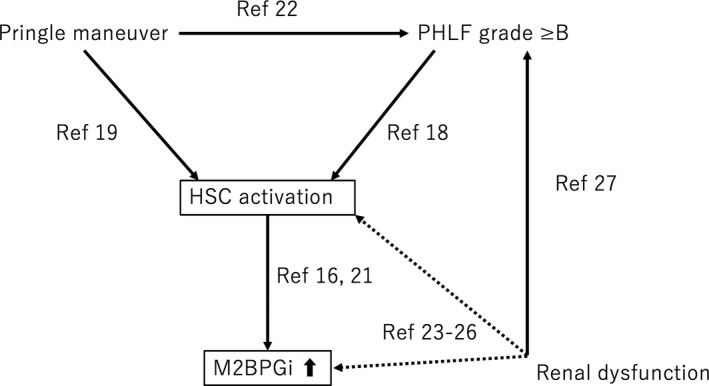
Summary of discussion why three factors influence the postoperative elevation of Mac‐2 binding protein glycosylation isomer (M2BPGi)

Bekki et al^16^ reported that hepatic stellate cells (HSCs) were the source of M2BPGi in subpopulations of liver‐derived cells. In addition, M2BPGi levels reflected the activation of HSCs during the progression of liver fibrosis.^16^ This may explain the rapid decrease of M2BPGi levels after patients with hepatitis C achieved a sustained virus response.^17^ Acute liver failure^18^ and ischemia‐reperfusion^19^ were reported to be associated with HSC activation. Following liver injury, HSCs become activated, trans‐differentiating from vitamin A‐storing cells to myofibroblasts, which are proliferative, contractile, inflammatory, and chemotactic, and which are characterized by enhanced extracellular matrix production.^20^ Activated HSCs in these situations may have a high potential to secrete M2BPGi. Indeed, it was reported that M2BPGi was increased in liver transplant patients with acute cellular rejection, even when fibrosis was absent.^21^ Furthermore, Morio et al^14^ reported that serum M2BPGi values were associated with clinical outcomes such as acute liver failure, development of hepatic coma, liver transplant, and death, suggesting that M2BPGi might reflect the severity of liver injury. In this study, the ΔM2BPGi ratio increased with advancing PHLF grade, which confirmed the previous study results. Liver resection with a longer total Pringle time causes greater ischemia‐reperfusion injury in the liver. Thus, prolonged total Pringle time and PHLF were likely to be associated with the activation of HSCs and secretion of M2BPGi, which continued for at least 1 month after liver resection. Furthermore, a recent study reported that total Pringle time was associated with PHLF (Figure 5).^22^

The current study also showed that the elevation of the postoperative value of M2BPGi was associated with preoperative creatinine. Few reports have referred to the relationship between M2BPGi and renal function. Okada et al^23^ reported elevated M2BPGi was associated with a decreased estimated glomerular filtration rate in chronic heart failure patients with abnormal liver function. Previous reports demonstrated that tumor necrosis factor‐α and interferon‐γ can increase M2BPGi expression in fibroblasts.^24^, ^25^ Patients with end‐stage renal failure were in systemic inflammatory status, as evidenced by increased levels of cytokines such as interleukin‐6 and tumor necrosis factor‐α.^26^ Thus, patients with renal failure might have a tendency to increase M2BPGi expression. In addition, renal failure was also reported to be a risk factor for PHLF (Figure 5).^27^

We had one patient whose M2BPGi markedly dropped after liver resection. Given her background liver was F0 fibrosis, her preoperative M2BPGi might be accidentally high. Further, none of the three factors, creatinine, PHLF or total Pringle time, were found in her. These combinations might be related to postoperative decrease of M2BPGi.

In this study, we examined M2BPGi values approximately 1 month after liver resection. Morio et al reported that serum M2BPGi values were increased in patients with acute liver injury and decreased following recovery. They defined “recovery” as a normalized serum aminotransferase value. The liver function of patients with PHLF in this study was damaged compared with patients without PHLF at 1 month after liver resection (data not shown), although serum aminotransferase values had normalized at that time. The M2BPGi value of patients with PHLF might decrease to the preoperative level thereafter. Thus, a longer follow up is needed to confirm this.

Why different serologic fibrosis markers exhibit different behaviors after liver resection is unclear. In our study cohort, the Δ4COL7S ratio exhibited a similar tendency to the ΔM2BPGi ratio regarding its relationship with PHLF. Type IV collagen is found primarily in the basal lamina, although the basal lamina comprised of type IV collagen appeared around the liver sinusoids after liver injury.^28^ Serum levels of 4COL7S, which has greater sensitivity and specificity for the detection of cirrhosis than type IV collagen, was increased abruptly following acute liver injury.^29^ It was also reported that type IV collagen accumulated in HSCs during acute viral hepatitis.^27^ Thus, the elevation of 4COL7S values after PHLF is reasonable and might reflect the activation of HSCs. Although hyaluronic acid is mostly secreted by HSCs,^30^ hyaluronic acid exhibited a different tendency from M2BPGi regarding its relationship with PHLF.^31^ Further study is required to confirm this.

The ΔM2BPGi ratio was significantly influenced by postoperative complications, such as complications of Clavien‐Dindo Class II or more and PHLF in the single linear regression. Due to short observation periods, we could not evaluate the impact of ΔM2BPGi ratio to patients’ prognosis. It has been reported that post‐hepatectomy complications were predictive of a worse overall survival in patients with HCC.^32^ Thus, ΔM2BPGi ratio might be associated with long‐term outcomes of patients who underwent liver resection.

A major limitation of this study was its observational design, which was exploratory in nature. The small subject number of the study and its single‐center‐based design limited the power of the explanation of results. Furthermore, the number of days after liver resection to postoperative serum sampling differed by individual. To minimize the influence of these factors, we excluded patients whose M2BPGi were measured more than 1 month after liver resection. We also confirmed that the ΔM2BPGi ratio was less affected by the number of days between operation and serum sampling by simple linear regression (Table 2). Finally, we did not determine the timely elevation of M2BPGi values during PHLF or liver resection because of the study design. Therefore, we should interpret the results of this study as predictors for the elevation of M2BPGi value at 1 month after liver resection, and not for any other time points.

In conclusion, preoperative creatinine, PHLF grade ≥B and total Pringle time were associated with the elevation of M2BPGi 1 month after liver resection. These observations emphasize the previous suggestion that M2BPGi is likely to reflect liver damage, liver inflammation, and renal function not limited to liver fibrosis.

## DISCLOSURE

Conflict of interests: Authors declare no Conflict of Interests for this article.

Author Contributions: 1) the conception or design of the work, or acquisition, analysis or interpretation of data for the work, Imai; Maeda; Wong; Sanefuji; Kayashima; Takeishi; Harada; Ikegami. 2) Drafting the work or revising it critically for important intellectual content; Imai; Yoshiya; Itoh. 3) Final approval of the version to be published, Maeda; Yoshizumi; Mori. 4) Agreement to be accountable for all aspects of the work in ensuring that questions related to the accuracy or integrity of any part of the work, Mori.
